# Recent efficacy of modified right vertical infra-axillary thoracotomy for ventricular septal defects in pediatric patients with younger age and Low body weight

**DOI:** 10.3389/fped.2025.1683641

**Published:** 2026-01-07

**Authors:** Heqi Zhang, Hua Cao, Weijie Liang, Taibing Fan

**Affiliations:** 1 Children's Heart Center, Fuwai Central China Cardiovascular Hospital, Zhengzhou, Henan, China; 2 Department of Cardiac Surgery, Central China Fuwai Hospital of Zhengzhou University, Zhengzhou, Henan, China

**Keywords:** infants, median sternotomy, modified right vertical infra-axillary thoracotomy, pulmonary function, ventricular septal defects

## Abstract

**Objective:**

To evaluate the safety and efficacy of a modified right vertical infra-axillary thoracotomy (MRVIAT) technique for treating ventricular septal defects (VSD) in infants younger than six months. and low body weight (≤5 kg).

**Methods:**

From January 2020 to December 2024, the study included 204 infants who underwent MRVIAT and 62 infants who underwent median sternotomy (MS). General, perioperative, and follow-up data were analyzed retrospectively.

**Results:**

Operation time, intraoperative bleeding, 24-h postoperative chest tube drainage, mechanical ventilation time, postoperative intensive care unit time, extubation rate within 24 h postoperatively, hospital costs, and incision length were shorter in the MRVIAT group than in the MS group (*P* < 0.05). Peak airway pressure, lung dynamic compliance (Cdyn), and oxygenation index were similar in both groups at preoperative and postoperative time points (*P* > 0.05). There were no deaths in either group, and there was no significant difference in complication rates (*P* > 0.05). At 60 months (with a median follow-up of 25 months), two cases of sternal deformity occurred in the MS group, but none occurred in the MRVIAT group.

**Conclusion:**

ventricular septal defect repair by MRVIAT in younger children with low body weight is less invasive. It provides good incision exposure, does not affect pulmonary function, reduces intraoperative bleeding and postoperative hospital stay and costs, and facilitates rapid recovery. This procedure is safe and effective and can be considered an alternative to median sternotomy.

## Introduction

Ventricular septal defect (VSD) is a common congenital heart defect (CHD). The median sternotomy (MS) approach has long been considered the gold standard for surgical repair and has produced favorable clinical outcomes (1, 2). However, the MS approach is associated with substantial surgical trauma, significant blood loss, prolonged recovery times, and an increased risk of postoperative thoracic deformities. These limitations have prompted cardiac surgeons to seek minimally invasive alternatives. Advances in surgical equipment and techniques have led to less invasive methods, such as right anterolateral thoracotomy and right vertical infra-axillary thoracotomy (RVIAT) (3–5). Nonrestrictive VSD often increases pulmonary blood flow, which makes it a common trigger for pneumonia in children. Pulmonary edema associated with VSD can lead to congestive heart failure (6). However, a weight of less than 5 kg and an age of less than 6 months are considered contraindications for early application of this approach in infants due to safety concerns. For example, postoperative respiratory insufficiency is a major concern because access to the intracardiac anatomy through the thoracic cavity can compress the fragile infant lung (7). With growing experience in RVIAT procedures, however, modifications to the technique have been developed that allow for improved surgical outcomes and greater safety for smaller infants. This study aims to retrospectively evaluate the safety, efficacy, and pulmonary function outcomes of MRVIAT in VSD infants of a younger age (≤6 months) and low body weight (≤5 kg) who are susceptible to pneumonia and delayed growth and development (8).

## Methods

From January 2020 to December 2024, a total of 266 infants underwent VSD repair at Central China Fuwai Cardiovascular Hospital, All procedures were performed by the same surgeon. Of these infants, 119 were male and 147 were female. The mean age was 3.2 ± 1.4 months, and the mean body weight was 4.5 ± 0.5 kg. The inclusion criteria were as follows: (1) body weight ≤5 kg and age ≤6 months, (2) completion of preoperative laboratory tests, chest x-ray, and electrocardiogram with VSD confirmed by echocardiography or CT angiography, (3) symptoms of growth retardation or recurrent pneumonia meeting VSD repair criteria, and (4) treatable simple cardiac anomalies during VSD repair. These anomalies included atrial septal defect, patent foramen ovale, patent ductus arteriosus, mitral or tricuspid regurgitation, aortic valve prolapse, and right ventricular outflow tract obstruction. Exclusion criteria: (1) pleural or respiratory diseases, (2) hematological disorders or multi-organ dysfunction, (3) complex cardiac anomalies including tetralogy of Fallot, and (4) diagnosed Eisenmenger syndrome. Of the 266 infants, 204 were assigned to the MRVIAT group and underwent VSD repair using the modified RVIAT approach. Meanwhile, 62 infants were assigned to the MS group and received repair via the MS approach. The study was approved by the Ethics Committee of Central China Fuwai Cardiovascular Hospital [Approval Number: (2024) Review No. 64], and written informed consent was obtained from the parents of all participants.

### Surgical technique

**Figure 1:**
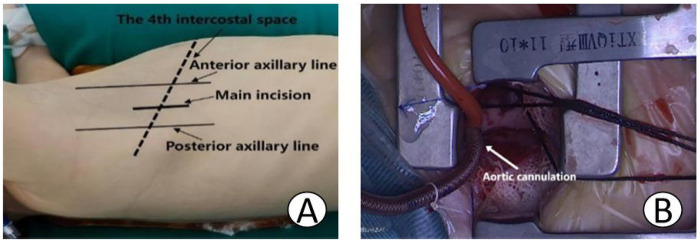
**(A)** Shows the right lateral decubitus position. **(B)** Illustrates the use of moist gauze for lung shielding and initiation of cardiopulmonary bypass.

**Figure 2:**
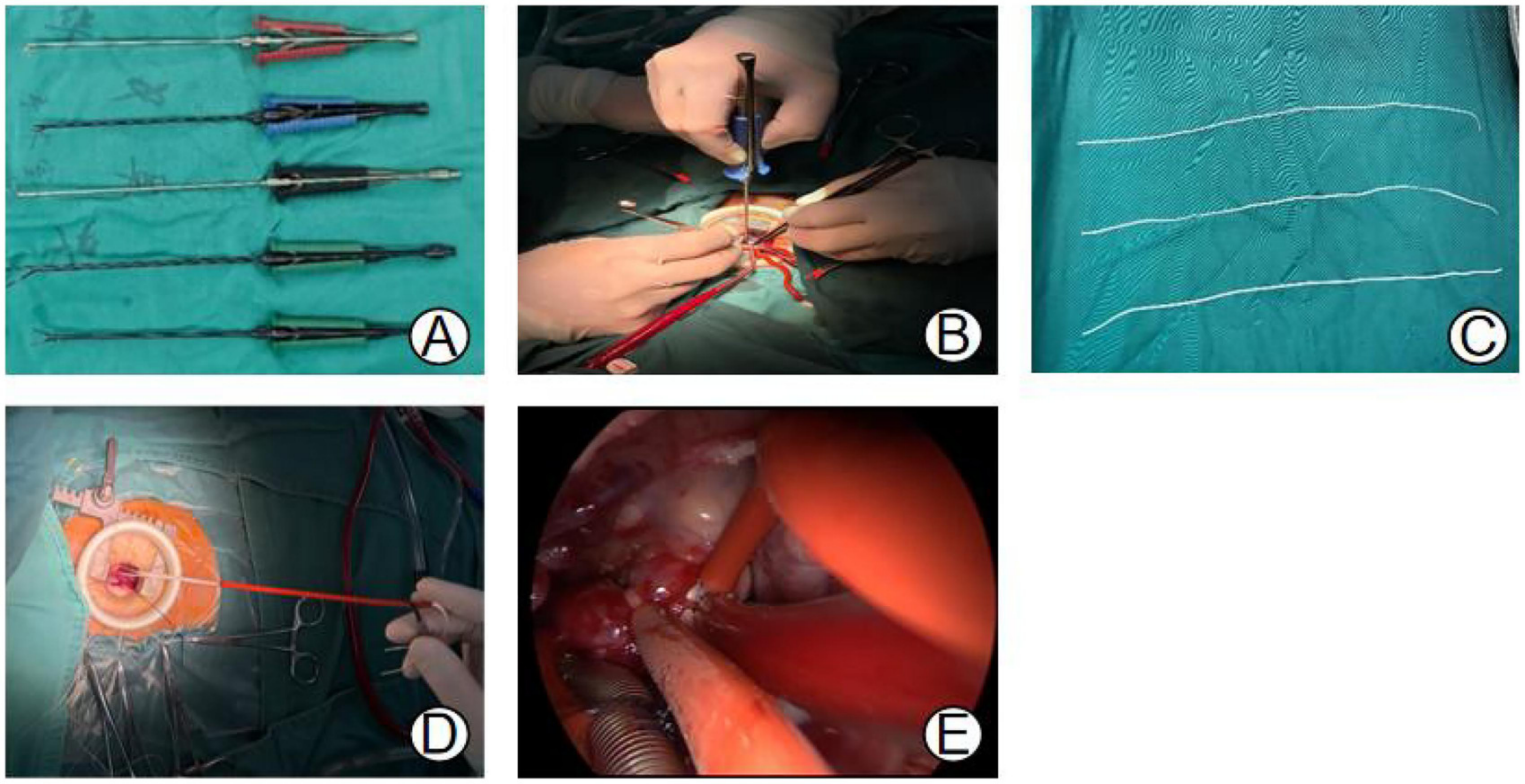
**(A,B)** Surgical procedures were performed intraoperatively using long-handled minimally invasive surgical instruments. **(C,D)** Aortic occlusion was achieved using aortic occlusion bands. **(E)** Aortic occlusion was performed by tightening the sleeve.

**Figure 3:**
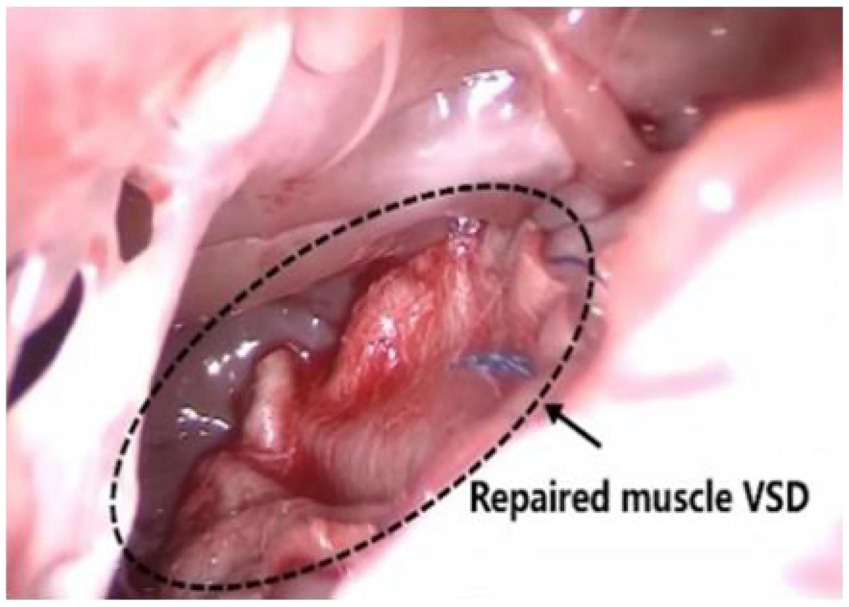
Shows the successful repair of a muscular ventricular septal defect.

**Figure 4:**
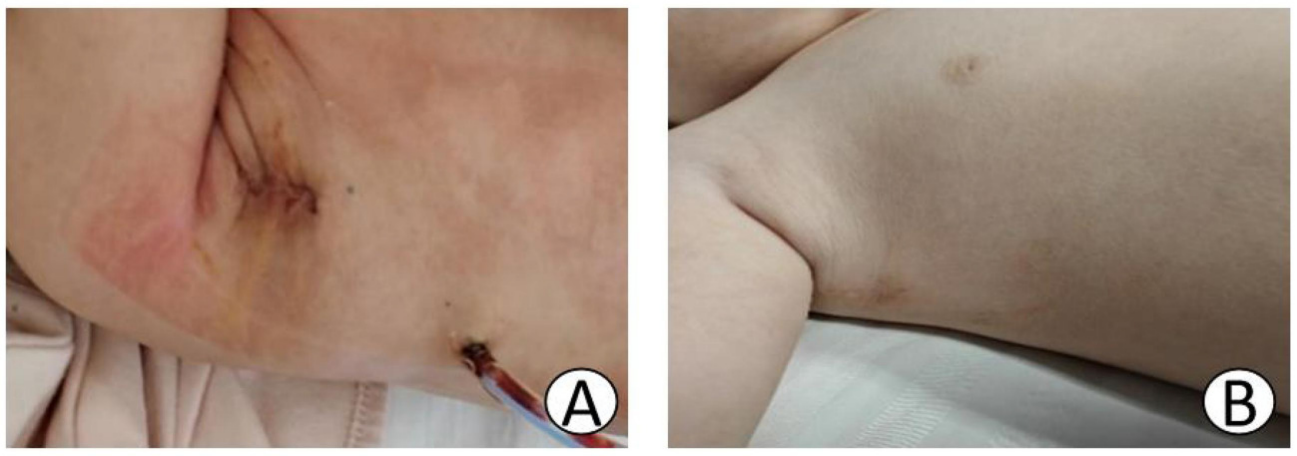
**(A)** Shows the surgical incision at the time of preparation for extubation on the second postoperative day. **(B)** The “invisible incision” 6 months postoperatively.

After receiving combined intravenous and inhalation anesthesia followed by tracheal intubation, pediatric patients in the MS group were placed in a supine position, and a midline thoracic incision measuring approximately 6–7 cm was made. The MRVIAT group was placed in the left lateral decubitus position with the right upper limb abducted and the elbow flexed. The limb was secured to the headrest to expose the axillary region (Figure 1A). After disinfection and draping, the right upper arm was released and allowed to rest naturally to avoid potential brachial plexus injury. Then, a longitudinal incision of approximately 1–2 cm was made at the fourth intercostal space along the right mid-axillary line. The subcutaneous tissue was then dissected, and a segment of the serratus anterior and intercostal muscles was incised. After temporary ventilation, the thoracic cavity was accessed via the fourth intercostal space, and a thoracic retractor was used to expand the operative field. The right lung was shielded with moist gauze to prevent potential damage. Next, a longitudinal pericardiotomy was performed 2 cm anterior to the right phrenic nerve. Then, pericardial suspension was performed at the sites of the superior vena cava, right atrium, inferior vena cava, and ascending aorta to expose the heart. Simultaneously, moist gauze was reapplied to protect the lungs (Figure 1B). The space between the aorta and pulmonary artery was then dissected to allow for the introduction of an occluding band (Figures 2C,D). After heparinization, purse-string sutures were carefully placed on the ascending aorta, as well as the superior and inferior venae cavae. Then, the following cannulae were inserted: aortic, right-angle metal venous, and inferior vena cava. Upon initiating cardiopulmonary bypass, the superior and inferior vena cava ligatures were tied to achieve vascular occlusion. After reducing the flow, the ascending aorta was occluded with an occluding band. Cardioplegic solution was then administered at the aortic root to induce cardiac arrest. Once cardiac arrest was satisfactory, the perfusion cannula was removed from the aortic root. The right atrium was incised and a left heart drainage catheter was inserted through the patent foramen ovale. The necessary surgical repairs were then performed using minimally invasive instruments (see Figures 2A,B). Subarterial and muscular VSD were visualized via the tricuspid approach (Figures 2B, 3). An appropriately sized bovine pericardial patch was selected for continuous suturing of the VSD. Several procedures were performed simultaneously, including Subaortic membrane resection, mitral and pulmonary valvuloplasty, and ligation of the patent ductus arteriosus. The patent foramen ovale was closed with continuous sutures, and circulation was restored after thorough ventilation. After clamping the aorta (Figure 2E), the heart spontaneously restarted with the restoration of sinus rhythm. After a period of parallel running, cardiopulmonary bypass was successfully discontinued and modified ultrafiltration was initiated. Prior to withdrawing cardiopulmonary bypass, transesophageal echocardiography confirmed the absence of residual shunting and aortic regurgitation. After weaning from cardiopulmonary bypass, gradually wean from the cardiopulmonary bypass circuit,closure of the right atrium, careful hemostasis was achieved, and a right chest tube was inserted into the sixth intercostal space. After lung reexpansion, the intercostal spaces were sutured. Finally, the subcutaneous tissue and skin were closed with absorbable sutures (Figure 4). The pediatric patients were then transferred to the intensive care unit (ICU) for further management.

### Pulmonary function assessment

Peak airway pressure (PIP) was measured preoperatively and at two time points postoperatively: immediately after surgery and two hours after surgery. Lung dynamic compliance (Cdyn) was also assessed at these intervals. It was calculated as tidal volume divided by the difference between peak inspiratory pressure and positive end-expiratory pressure (PEEP), and was then normalized to body weight (Cdyn/body weight). The oxygenation index (OI) was determined by dividing the arterial oxygen partial pressure by the inspired oxygen concentration.

### Statistical methods

Statistical analyses were performed using SPSS version 26.0. Data that were normally distributed were expressed as the mean ± standard deviation (x ± s) and were compared between groups using an independent *t*-test. Non-normally distributed data were expressed as the median (interquartile range) [M(Q1, Q3)] and analyzed using the Mann–Whitney *U*-test. Categorical variables were analyzed using the chi-square test. Statistical significance was defined as *P* < 0.05, with an alpha level of 0.05.

### Baseline characteristics

There were no statistically significant differences between the groups in gender distribution, age, body weight, VSD diameter, VSD type, incidence of preoperative pneumonia, mechanical ventilation rate, preoperative STAMP score (low-to-moderate risk: 0–3, high risk: ≥4 requiring nutritional intervention, severe risk: ≥7 requiring clinical nutritionist consultation), presence of associated cardiac anomalies, preoperative pulmonary hypertension, or valvular regurgitation and stenosis (*P* > 0.05). Details are shown in Table 1.

**Table 1:** Comparison of baseline characteristics between the two groups.

Variables	MRVIAT (*n* = 204)	MS (*n* = 62)	*P*
Gender (female/*n*)	87.0 (42.6%)	32.0 (51.6%)	0.214
Age (m)	3.0 ± 1.4	3.7 ± 1.4	0.062
Weight (kg)	4.5 ± 0.5	4.5 ± 0.5	0.833
Pre-LVEF, (%)	68.3 ± 4.3	67.6 ± 4.4	0.497
History of severe preoperative pneumonia, *n* (%)	78.0 (38.2%)	22.0 (35.5%)	0.695
Preoperative Mechanical ventilation, *n* (%)	43.0 (21.1%)	16.0 (25.8%)	0.432
STAMP, *n* (%)	3.5 ± 0.7	3.5 ± 0.6	0.266
Perimembranous VSD, *n* (%)	174 (85.3%)	52 (83.9%)	0.784
Doubly committed subarterial VSD, *n* (%)	24 (11.8%)	8 (12.9%)	0.809
Muscular VSD, *n* (%)	3 (1.5%)	1 (1.6%)	0.936
Perimembranous and Muscular VSD, *n* (%)	3 (1.5%)	1 (1.6%)	0.936
VSD size, *n* (%)	8.3 ± 1.9	9.8 ± 1.8	0.233
Concomitant repair of two or more defects			
Atrial septal defect repair, *n* (%)	65 (31.9%)	24 (38.7%)	0.317
Mitral valve repair, *n* (%)	2 (1.0%)	1 (1.6%)	0.550
Patent foramen ovale closure, *n* (%)	91 (44.6%)	36 (58.1%)	0.063
Patent ductus arteriosus ligation, *n* (%)	7 (3.4%)	3 (4.8%)	0.703
Pulmonary arterioplasty, *n* (%)	2 (1.0%)	1 (1.6%)	0.550
Right ventricular muscle bundle resection, *n* (%)	5 (2.5%)	1 (1.6%)	0.697
Subvalvular aortic membrane resection, *n* (%)	10 (4.9%)	3 (4.8%)	0.255
Moderate or Severe pulmonary hypertension, *n* (%)	126 (61.8%)	38 (61.3%)	0.946
Preoperative mitral valve regurgitation moderate or severe, *n* (%)	3 (1.5%)	2 (3.2%)	0.373
Preoperative tricuspid valve regurgitation moderate or severe, *n* (%)	2 (1.0%)	1 (1.6%)	0.550
Preoperative pulmonary valve stenosis moderate or severe, *n* (%)	2 (1.0%)	1 (1.6%)	0.550

STAMP, screening tool for the assessment of malnutrition in paediatrics; LVEF, left ventricular ejection fraction.

### Perioperative outcomes

Compared to the MS group, the MRVIAT group had significantly shorter operation and postoperative mechanical ventilation times, a higher 24-h extubation rate, shorter postoperative ICU stays, less intraoperative blood loss, lower postoperative 24-h thoracic drainage volumes, shorter postoperative hospital stays, lower hospital costs, and smaller incisions (*P* < 0.05). There were no significant differences in cardiopulmonary bypass (CPB) time, aortic cross-clamp (ACC) time, or postoperative left ventricular ejection fraction (LVEF) between the groups (*P* > 0.05). Detailed results are shown in Table 2.

**Table 2:** Comparison of perioperative results between the two groups.

Variables	MRVIAT (*n* = 204)	MS (*n* = 62)	*P*
Operation time, min	132.3 ± 11.1	151.9 ± 20.2	<0.001
Cardiopulmonary bypass time, min	53.6 ± 11.8	65.8 ± 18.1	0.242
Aortic cross-clamp time, min	32.4 ± 8.9	41.2 ± 12.6	0.285
Postoperative hospital stay time, days	8.5 ± 2.0	12.9 ± 1.5	0.021
Postoperative mechanical ventilation time, h	8.0 (6.0,13.0)	15.5 (12.0,28)	<0.001
Postoperative intensive care unit stay time, days	2.9 (2.0,3.7)	2.9 (2.5,3.8)	0.036
Postoperative 24-h thoracic drainage volume, ml	11.3 ± 1.2	14.3 ± 1.6	0.004
Intraoperative blood loss, ml	11.6 ± 1.1	15.8 ± 2.0	0.000
Postoperative LVEF, %	67.1 ± 7.2	66.5 ± 4.1	0.107
Postoperative early extubation within 24 h, *n* (%)	68 (33.3%)	12 (19.4%)	0.036
Hospitalisation costs, ten thousand CNY	7.0 ± 0.7	7.8 ± 0.4	<0.001
Incision length, cm	1.7 ± 0.2	8.3 ± 0.3	<0.001
Complications			
Residual shunt, *n* (%)	2 (1.0%)	1 (1.6%)	0.550
Arrhythmia, *n* (%)	2 (1.0%)	2 (3.2%)	0.203
Postoperative valve stenosis, *n* (%)	0	0	
Incision infection, *n* (%)	0	2 (3.2%)	0.054
Reoperation, *n* (%)	0	1 (1.6%)	0.233
Neurological complication, *n* (%)	0	0	
Pneumothorax, *n* (%)	0	0	
Pulmonary infection, *n* (%)	1 (0.5%)	1 (1.6%)	0.413
Low cardiac output syndrome, *n* (%)	0	0	
Chest deformity, *n* (%)	0	3 (4.8%)	0.012
Pleural effusion, *n* (%)	0	0	
Atelectasis, *n* (%)	1 (0.5%)	1 (1.6%)	0.413
Dead, *n* (%)	0	0	

LVEF, left ventricular ejection fraction.

### Postoperative complications

Successful surgical repairs were achieved in both groups, with no significant difference in complication rates (*P* > 0.05). One patient in the MS group required re-thoracotomy due to cardiac tamponade caused by excessive bleeding. Postoperative arrhythmias occurred in two patients in both the MS and MRVIAT groups; these resolved with electrolyte replacement or antiarrhythmic therapy. Both groups experienced minimal residual shunting that resolved spontaneously within three to 12 months of follow-up. There were no incision infections or thoracic deformities observed in the MRVIAT group. However, three cases of thoracic deformity occurred in the MS group (two cases of pectus carinatum and one case of pectus excavatum). This difference was statistically significant (*P* < 0.05). Rates of incision infection, pulmonary infection, and atelectasis were comparable between the groups (*P* > 0.05; see Table 2).

### Pulmonary function comparison

There was no statistically significant difference in PIP and Cdyn between the preoperative, immediate postoperative, and 2-h postoperative periods in the MRVIAT group compared with the MS group (*P* > 0.05). Similarly, there was no significant difference in OI between the preoperative, immediate postoperative, and 24-h postoperative periods in the MRVIAT group compared with the MS group (*P* > 0.05). See Table 3.

**Table 3:** PIP, cdyn and OI before and after operation were compared between the two groups.

Variables	MRVIAT (*n* = 204)	MS (*n* = 62)	*P*
PIP(cmH2O)			
Pre-operation/(x ± s, cmH2O)	16.1 ± 1.1	16.2 ± 1.1	0.206
Immediate postoperative/(x ± s, cmH2O)	19.0 ± 1.3	19.1 ± 1.2	0.744
2 h postoperative/(x ± s, cmH2O)	16.8 ± 1.0	16.8 ± 1.2	0.201
Cdyn			
Pre-operation/(x ± s)	0.76 ± 0.13	0.77 ± 0.19	0.073
Immediate postoperative/(x ± s)	0.75 ± 0.13	0.74 ± 0.15	0.601
2 h postoperative/(x ± s)	0.76 ± 0.14	0.74 ± 0.17	0.204
OI(mmHg)			
Pre-operation/[M(Q1, Q3), mmHg]	255.0 (162.0, 356.8)	238.5 (162.3, 353.8)	0.863
Immediate postoperative/[M(Q1, Q3), mmHg]	226.0 (145.0, 339.3)	230.0 (143.3, 328.3)	0.761
24 h postoperative/[M(Q1, Q3), mmHg]	228.0 (168.0, 394.0)	262.0 (186.8, 379.0)	0.423

### Follow-up

Patients were seen in the outpatient clinic or contacted by telephone for follow-up. Echocardiographic findings were routinely reviewed at one, three, six, and 12 months postoperatively, and annually thereafter. Echocardiographic data were available for 186 (91.2%) and 58 (93.5%) patients in the MRVIAT and MS groups, respectively. This included 186 (91.2%) and 58 (93.5%) patients at discharge, as well as 167 (81.9%) and 53 (85.5%) patients in the MRVIAT and MS groups six months or more after discharge. The median follow-up period was 15 months (range, 1–52 months). During follow-up, no thoracic deformities or valve regurgitation related to surgery were found in the MRVIAT group. Three sternal deformities were found in the MS group (*P* < 0.05); two were treated with surgery, and one is under observation.

## Discussion

Malnutrition is common in hospitalized children with CHD, especially when combined with severe pneumonia, heart failure, and pulmonary hypertension. When hemodynamic parameters are drastically altered, it can lead to severe malnutrition; these children often urgently need surgical treatment to relieve the cardiac burden. Advancements in surgical techniques and medical equipment for CHD have led to successful surgical procedures. After the public began to favor minimally invasive surgical incisions, surgeons started exploring this approach. The right anterolateral incision, the right straight axillary incision, and the right posterior lateral incision were developed successively (3–5). However, the right anterolateral incision can lead to complications in pubertal breast development and peripheral extracorporeal circulation. Thus, the RVIAT is increasingly preferred (4, 5). Ventricular septal defect (VSD), a common CHD with a high prevalence, has been shown to be safe and effective for surgical repair with an RVIAT (9, 10).

These infants have a history of recurrent pneumonia and developmental arrest, and they tend to have a combination of moderate to severe pulmonary hypertension. They require urgent surgery for unrestricted VSD. However, due to safety concerns, infants weighing less than 5 kg are considered contraindicated for early use of this approach. Postoperative respiratory failure, for example, is a major concern because accessing the intracardiac anatomy through the chest can compress an infant's immature lungs. A 2015 report confirmed that the RVIAT in infants under 5 kg could achieve the same surgical outcomes as a MS and would not affect postoperative lung function. However, it is worth noting that they used an RVIAT, which may affect breast development in female patients (11, 12), and the incision is relatively long. They reported a mean incision length of 5.99 ± 0.86 cm, which increases scarring as the child grows and can lead to unsightly results. In this study, our MRVIAT incision greatly reduced the length of the incision. The incision was hidden; none of the incisions in the MRVIAT group were more than 2 cm in length. The incision was made vertically close to the axilla. This did not interfere with the development of the child's mammary glands and provided the most hidden incision. No small vertical right axillary incision is hidden.

The MRVIAT technique is based on RVIAT, which optimizes surgical instruments for deep and delicate operations. This results in a small incision without interfering with the operator's work. The main improvements include the following: (1) Use of long-handled surgical instruments. These instruments allow us to perform deep and delicate operations such as repairing muscular and subxiphoid septal defects through the tricuspid valve route. (2) Modified aortic cross-clamping technique. First, the aortopulmonary interval is dissected, and then the aortic cross-clamp tape is passed through this interval. This method avoids the disadvantage of traditional occlusion forceps occupying the surgical field, significantly saving surgical space. The MRVIAT has many advantages: (1) The incision is minimally invasive. It is ultra-small, vertical, and immediately adjacent to the axilla, providing significant concealment. (2) There is no post-operative breast or sternum deformity. The incision does not require sternum separation and does not affect breast development because it does not injure breast-related tissues, unlike the anterolateral incision. (3) It has a short learning curve and is easy to master. For cardiac surgeons specializing in CHD, it does not change their operating habits, making it popular.

According to previous studies (7, 13), the RVIAT approach does not adversely affect postoperative lung function in children. While the MS avoids potential impact on lung tissue, intraoperative bleeding, trauma, and disruption of thoracic stability significantly impact postoperative recovery and thoracic deformity in children. In contrast, the RVIAT approach preserves thoracic integrity and respiratory dynamics. Furthermore, a preoperative or postoperative nerve block of the anterior serratus muscle reduces postoperative pain in children. In this study, comparing the two groups' perioperative peak airway pressure, Cdyn, and oxygenation index revealed that the MRVIAT approach does not lead to a deterioration in lung function in infants. Additionally, the time spent on postoperative mechanical ventilation, in the ICU, and the postoperative hospital stay indirectly suggest that pulmonary dysfunction did not occur after RVIAT.

This study found that the cardiopulmonary bypass time and aortic cross-clamp duration for the MRVIAT approach were similar to those for the MS approach. However, the MRVIAT group had a significantly shorter operation time than the MS group (*P* < 0.05). This is because the MRVIAT approach eliminates the need for complex procedures such as sternum splitting, closure, and associated hemostatic maneuvers. Only a section of the intercostal muscles was incised, which resulted in reduced intraoperative blood loss and decreased 24-h postoperative drainage compared to the MS approach. Furthermore, the MRVIAT group had shorter durations of postoperative ventilator support, ICU stay, and length of postoperative hospitalization, as well as shorter incision length, than the MS group. Additionally, hospitalization costs were lower in the MRVIAT group. Therefore, for infants with VSD weighing ≤5 kg and aged ≤6 months, the MRVIAT appears to be a favorable alternative to the MS approach.

Initially, the choice of indications for the MRVIAT approach in younger infants is limited to simple VSD, which may be combined with atrial septal defects or patent foramen ovale. In our study, we performed surgical repair on younger infants with low body weight using a small, straight incision of no more than 2 cm. We completed repairs of a wide range of combined malformations, such as right ventricular outflow tract sparing, subaortic diaphragm resection, valvuloplasty, and stenosis, as well as ductus arteriosus ligation, all within the same time period. To improve the operative space, we used a cannula one size smaller than the standard cannula to establish extracorporeal circulation, increasing the negative pressure to improve vena cava drainage. Some scholars have also placed the inferior vena cava cannula through the sixth intercostal space or percutaneously, using it to place a chest tube after surgery (3, 14). In the MRVIAT group, we also applied this technique.

This study shows that MRVIAT achieves similar efficacy to MS and has clear advantages over MS access in terms of intraoperative bleeding, postoperative recovery, and the incidence of long-term sternal deformity. Regarding postoperative complications and long-term follow-up, no statistically significant differences were observed between the two groups in terms of residual shunts, arrhythmia, reoperations, pulmonary atelectasis, or pulmonary infections. However, the incidence of postoperative incisional infections and sternal deformities was lower in the MRVIAT group than in the MS group (two cases of chicken chest and one case of funnel chest).

## Conclusion

In conclusion, the advantages of the MRVIAT technique for treating children with VSD who are younger than six months old and weigh less than five kilograms and require urgent surgery include minimal trauma, good thoracic stability, rapid postoperative recovery, and lower hospital costs. Additionally, the MRVIAT technique avoids the influence of the anterolateral incision on the development of the pectoral muscles and mammary glands, making it particularly suitable for girls and an alternative to median sternotomy.

## Limitations

This study has several limitations. First, it was a retrospective data analysis. Second, there was a lack of data on the degree to which infants were more comfortable, which remains difficult to measure. Third, the follow-up time was insufficient; longer follow-up is needed to draw stronger conclusions.

## Data Availability

The raw data supporting the conclusions of this article will be made available by the authors, without undue reservation.
